# Embedding operational research into national disease control programme: lessons from 10 years of experience in Indonesia

**DOI:** 10.3402/gha.v7.25412

**Published:** 2014-10-23

**Authors:** Yodi Mahendradhata, Ari Probandari, Bagoes Widjanarko, Pandu Riono, Dyah Mustikawati, Edine W. Tiemersma, Bachti Alisjahbana, on behalf of the Tuberculosis Operational Research Group (TORG)

**Affiliations:** 1 Center for Health Policy and Management, Faculty of Medicine, Universitas Gadjah Mada, Yogyakarta, Indonesia; 2 Faculty of Medicine, Institute of Public Health, University of Heidelberg, Heidelberg, Germany; 3 Department of Public Health, Faculty of Medicine, Sebelas Maret University, Surakarta City, Indonesia; 4 Department of Health Education and Behavioral Science, Faculty of Public Health, Diponegoro University, Semarang, Indonesia; 5 Department of Biostatistics and Population, Faculty of Public Health, University of Indonesia, Depok, Indonesia; 6 National Tuberculosis Control Program, Ministry of Health, Jakarta, Indonesia; 7 KNCV Tuberculosis Foundation, The Hague, The Netherlands; 8 Department of Internal Medicine, Medical Faculty, Padjadjaran University, Bandung, Indonesia; 9 TB-HIV Research Center, Dr Hasan Sadikin Hospital, Bandung, Indonesia

**Keywords:** operational research, tuberculosis, disease control, policy, capacity building

## Abstract

There is growing recognition that operational research (OR) should be embedded into national disease control programmes. However, much of the current OR capacity building schemes are still predominantly driven by international agencies with limited integration into national disease control programmes. We demonstrated that it is possible to achieve a more sustainable capacity building effort across the country by establishing an OR group within the national tuberculosis (TB) control programme in Indonesia. Key challenges identified include long-term financial support, limited number of scientific publications, and difficulties in documenting impact on programmatic performance. External evaluation has expressed concerns in regard to utilisation of OR in policy making. Efforts to address this concern have been introduced recently and led to indications of increased utilisation of research evidence in policy making by the national TB control programme. Embedding OR in national disease control programmes is key in establishing an evidence-based disease control programme.

The importance of operational research (OR) for improving programmes and services has become increasingly recognised by the global health community (1). OR is considered to be essential for developing a strong knowledge base and identifying innovative strategies to improve performance of disease control programmes by improving patient care and the prevention and management of diseases (2, 3). Thus, OR should be an integral part of national disease control programmes in low- and middle-income countries (LMICs). Substantial funding for OR has been made available through mechanisms such as the Global Fund to Fight AIDS, Tuberculosis, and Malaria (GFATM). However, despite the interest in and recognition of its value, relatively little OR is being implemented within national disease control programmes in LMICs (1). This may be due to insufficient capacity and skills to design, undertake, analyse, and write up publication manuscripts adequate for publication in peer-reviewed international journals.

There have actually been many health research capacity building programmes developed for LMICs. The American Thoracic Society Methods in Epidemiologic, Clinical and Operations Research (ATS MECOR) programme began in Latin America as the International Respiratory Epidemiology course in 1994 and, in its 18th year, has developed into a five-level programme with a range of competencies (4). In the late 1990s, KNCV Tuberculosis Foundation set up a dedicated research unit to complement the work of the general technical assistance consultants, providing the evidence-base for TB control through capacity building, ranging from on-the-job training to full international PhD programmes (5). Programmes specifically aiming to strengthen OR capacity have also been developed and organised in the past years (1). The International Union Against Tuberculosis and Lung Disease (The Union) and the Centers for Disease Control and Prevention, Atlanta, Georgia, USA, have for many years organised short training programmes on OR. The International Tuberculosis Training Course in Japan has been running between 2001 and 2007, with 28 participants developing OR projects. Since 2009, the Union and Médecins Sans Frontières (MSF) collaborated to organise three module OR courses. More recently, WHO-TDR collaborated with the Union and MSF to develop the Structured Operational Research and Training Initiative (SORT-IT) (6).

We applaud the OR capacity building initiatives illustrated above. However, we note that these reputable initiatives are still predominantly driven by international agencies, with limited embedding into national disease control programmes. OR is best prioritised, designed, implemented, and replicated from within national disease control programmes (7, 8). We argue that this approach should become the norm for OR capacity building particularly in resource-constrained settings. Here, we present lessons based on our 10 years of experience embedding OR into the National TB Control Programme (NTP) in Indonesia.

## Embedding OR in Indonesia's NTP

Indonesia has a strong foundation in research, with a record of conducting OR before implementing a new strategy, such as short-course chemotherapy, DOTS including MDR-TB and TB/HIV management and hospital DOTS linkage (9–11). Preparatory activities were already underway in 2003 to establish the Tuberculosis Operational Research Group (TORG) with technical support from the Research Unit of KNCV Tuberculosis Foundation, supported by USAID. TORG was then officially established by a decree of the Directorate General of Disease Control and Environmental Health in 2004.

TORG members are experienced researchers from reputable public and private universities in Indonesia as well as representatives from the NTP and the National Institute of Health Research and Development. The official tasks of TORG are: 1) to provide technical assistance for conducting OR at the provincial/district level; 2) to facilitate capacity building for conducting and utilising OR at provincial level through intensive courses; 3) to provide review on relevance of independent OR proposals submitted to the national TB programme; and 4) to provide recommendations for evidence-based improvement of the national TB programme based on OR. A secretariat for TORG is provided within NTP.

Subsequently, since 2005, there has been an extensive scaling up of OR activities facilitated by TORG. These activities included: a district TB financing study providing evidence on the need for increased budget commitment at local level; a pilot study of TB-HIV surveillance which provided basis for a national TB-HIV policy; a study on TB case management in hospitals and the economic evaluation on public–private mix schemes strengthening evidence-based planning of network enforcement including all healthcare providers; and evaluation of intensified introduction of Xpert MTB/Rif in Indonesia. The increase in research activities is partly the result of high financial commitment achieved through wide donor support, notably DFID, USAID, and GFATM. Key to the success in the increase in research activities has been the participation of a broad range of national and international partners in conducting research and providing technical assistance. TORG has also made efforts to better align OR projects with NTP priorities by formulating the national OR agenda and organising call for proposals on behalf of NTP.

**Fig. 1:**
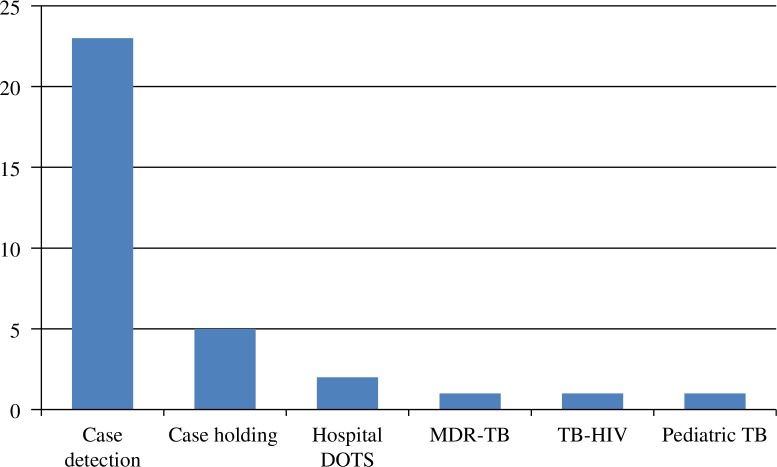
Topics covered by OR projects completed by participants of the intensive courses (*N*=33).

In parallel to supporting independent strategic OR projects illustrated above, TORG has organised intensive OR courses which have been designed based on the two-volume book entitled *Designing and Conducting Health System Research Project* (12, 13). The first course was facilitated by international experts in OR of KNCV and the Royal Dutch Tropical Institute (KIT), and co-facilitated by TORG members. In subsequent courses, TORG served as the lead course faculty. These courses have now been provided to eight batches of provincial teams from all over Indonesia. Each batch consisted of four provincial teams, generally composed of five participants (three from academic institutes; two TB programme staff members). The course has been designed to be conducted over a timeframe of 1.5 years consisting of a proposal development workshop (10–14 days), OR project implementation (6–12 months), a data analysis and report writing workshop (7–10 days), and result dissemination to the key stakeholders. The priority problem for each of the OR projects was chosen after intensive discussions between TORG course facilitators and locally relevant programme staff during a preparatory meeting before the start of the course in the province. Fig. 1 presents the topics of the OR projects conducted by the intensive course participants. More recently, representatives of these trained teams have been invited to participate in supplementary workshops for database management training, writing manuscripts for publication in international peer-reviewed journals, and intervention design. These supplementary workshops respond to needs which have not been addressed adequately in the original design of the intensive course. In order to improve utilisation of OR in policy making, recently, TORG has emphasised engaging policy makers through organising dissemination meetings and a workshop for writing policy briefs.

TORG's post training evaluation revealed that participants considered the OR courses to be excellent, providing them with focused training which were much needed to master the essential OR skills. They also reported that the training had motivated them to pursue more postgraduate education and inspired them to improve their local TB programme with innovative approaches. They highlighted as well, however, some challenges related to the training, including managing time (all of them had other duties) and publication writing (limited capacity for academic English writing).

Up to 2013, a total of 56 OR projects have been conducted, of which 46 were successfully completed (33 by participants of the intensive workshops) and 35 have been disseminated to key stake holders (Table 1). The OR projects which have not been disseminated were mainly those conducted at the earlier years when dissemination efforts were not yet systematically facilitated. Some of the dissemination meetings have produced written commitments from policy makers to follow up recommendations from the studies. TORG and NTP have also successfully organised four national TB research parades (2009, 2010, 2013, and 2014) to disseminate TB OR findings, and published a compilation of TB OR results. Furthermore, several OR studies supported by NTP have been presented at international conferences. Nine OR teams have attempted to write the manuscript. Four of these teams managed to complete the manuscripts and eventually published after subsequent revisions and resubmissions (Box 1). At least four doctoral theses partially building on OR projects facilitated through TORG have been successfully defended in reputable European universities (Table 2). TORG members have also been actively engaged in writing national strategies for TB control, national action plans, and national proposals to GFATM.

**Table 1:** Dissemination and follow up of completed OR projects (2003–2013)

	Projects facilitated through intensive course	Independent projects
Project initiated	33	23
Project completed	23	23
Disseminated and policy brief	22	13
Published in research compilation book	22	5
Submitted to international journal	4	2
*Published in international journal*	*4*	*–*
Followed by subsequent OR	4	–

**Table 2:** Doctoral thesis based on OR projects facilitated by TORG

Title		Year defended	University
Tuberculosis in Indonesia, host response and patient care	Alisjahbana B	2007	Radboud Universiteit, Nijmegen, the Netherlands
Piloting new interventions for tuberculosis control in Indonesia	Mahendradhata Y	2009	Ghent University, Ghent, Belgium
Revisiting the choice: to involve hospitals in the partnership for tuberculosis control in Indonesia	Probandari A	2010	Umea University, Umea, Sweden
Improving tuberculosis case finding in Indonesia	Ahmad R	2011	Erasmus MC, Rotterdam, the Netherlands

Box 1 **OR projects published in international journals**

1. Putra IW, Utami NW, Suarjana IK, Duana IM, Astiti CI, Putra I, et al. Factors associated to referral of tuberculosis suspects by private practitioners to community health centres in Bali Province, Indonesia. BMC Health Serv Res 2013; 13: 445.
2. Rintiswati N, Mahendradhata Y, Suharna S, Susilawati S, Purwanta P, Subronto Y, et al. Journeys to tuberculosis treatment: a qualitative study of patients, families and communities in Jogjakarta, Indonesia. BMC Public Health 2009; 9: 158.
3. Sakundarno M, Nurjazuli N, Jati SP, Sariningdyah R, Purwadi S, Alisjahbana B, et al. Insufficient quality of sputum submitted for tuberculosis diagnosis and associated factors, in Klaten district, Indonesia. BMC Pulm Med 2009; 9: 16.
4. Wahyuni CU, Budiono, Rahariyani LD, Sulistyowati M, Rachmawati T, Djuwari, et al. Obstacles for optimal tuberculosis case detection in primary health centers (PHC) in Sidoarjo district, East Java, Indonesia. BMC Health Serv Res 2007; 7: 135.

Within the period of 2003–2013, TORG successfully worked through successions of four Ministers of Health and four NTP managers. All four NTP managers were in general appreciative of the need for research evidence to support programme improvement. The current NTP manager in particular is highly engaged with TORG, for instance, in conduct of recent studies of strategic importance such as a survey of TB case management practices among private practitioners, implementation of Xpert MTB/RIF in five sites in Indonesia, a national TB prevalence survey, and estimates of TB prevalence. These studies have informed current policies of NTP. The current NTP management also promotes dissemination of OR activities through supporting presentations at international conferences, providing funding for publication in open access journals, and supporting submission to competitive calls for proposals (e.g. TB REACH).

Though TORG collaborates directly with the NTP, it should be noted that Indonesia's health system is highly decentralised, making collaboration with public health authorities at provincial and district level necessary. Accordingly, TORG's approach in capacity building has been based on training and mentoring of provincial district teams. The impact of TORG within policy networks across districts, beyond written commitments on changing policies and/or practices by policy makers signed during dissemination meetings, however, remains to be documented.

Recent results from Joint External Monitoring Mission conducted in 2011 and 2013 (unpublished) highlighted current challenges for TORG, noting: 1) limited impact on the performance of the TB control programme; 2) mechanism to propose, initiate, and conduct OR research has not been integrated and implemented within day-to-day programmatic activities in the provinces and districts despite the training; 3) OR findings have not been used optimally for policy development; 4) conducting OR is a part-time job, because investigators typically have other programmatic or academic duties; and 5) important programmatic issues such as reasons for low notification of previous treatment history and decreasing treatment success rate in large facilities have not yet been investigated.

TORG's activities have received financial support from USAID through various channels. USAID funding between 2004 and 2005 has been channelled through TBCTA (TB Coalition for Technical Assistance). This was continued between 2005 and 2010 through TB CAP (TB Control Assistance Program). Since 2011, funding has been channelled through TB CARE I. Moreover, TORG has received some financial support for courses and projects through Global Fund Round 8 which has been available since 2009. TORG has also received technical assistance from KNCV Tuberculosis Foundation (including mentoring) since its establishment in 2004 and has continued until the time of writing.

## Discussion

We have demonstrated that it is possible to actually embed OR into a national disease control programme, leading to long-term capacity building effort across the country. The main funding sources until now however are still from international agencies. It will be challenging to find local funding in the near future to sustain TORG and its activities in the long run. Long-term financial challenges are apparently also faced by other OR capacity building initiatives (6, 14, 15). There is thus a need to consider strategies for sustainability, for example, cost management and resource mobilisation.

Notably, the Ministry of Health has formulated an exit strategy from grants of the GFATM (16). Specific exit strategy for OR financing however still needs to be delineated. The budget of the national strategy for TB control 2010–2014 (17) indicates that less than 0.5% is allocated to promoting research and information. This suggests that there is still considerable effort to be made for increasing the funding allocation of OR in the budget of the national strategy for TB control in the upcoming period. The new Indonesian Health Fund which has recently been established by local philanthropists and the mounting push for the Indonesian Science Fund provides a window of opportunity for tapping into local funding sources (18). Content wise, there are limitations to the original OR course design. The number of international peer-reviewed publications also remains low and evidence of the impact of OR capacity building on programme performance has been limited. These concerns merit more discussions leading toward distillations of lessons learned for enforced OR capacity building in LMICs.

The intensive OR course was originally designed in 2004 based on a two-volume main reference (12, 13). The approach subsequently needed to be refined and adjusted based on emerging needs and observed limitations, which encompass data management, data analysis, and intervention design. These have been subsequently addressed through supplementary workshops. The Union's three module design, inserting a database management module between proposal completion and study conduct, has been demonstrated to be effective in terms of producing scientific publications (1, 3) and merits consideration for adoption in contexts where resources permit.

Scientific publications have been touted as one of the key performance indicators of OR (19). Thus, the low number of international peer-reviewed publications caused concerns. The limited number of scientific publications stemming from OR in various settings has also been reported from other OR courses (19). The main reasons for failure to produce manuscripts and publications documented from other OR courses include: wrong choice of research question, poorly designed studies resulting in weak results, inadequate writing and language skills, lack of dedicated time and opportunity for writing, peer-review rejection fatigue, no ethics clearance or exemption, rapid staff turnover, disapproval from supervisors, and lack of funding and infrastructure (19). TORG faced many of those factors as well, but the limited capacity in academic English-writing skills obviously was an important factor in our case. Academic English writing courses are hardly offered at universities and other higher education institutions in the country. Providing such courses arguably goes beyond the mandate of research organisations under national disease control programmes such as TORG. There could be potential leverage, however, through other more feasible complementary strategies, for example, publication writing workshops and mentorship.

The success in terms of scientific publications, however, arguably is also influenced by the heterogeneity of participants’ aptitude and abilities (e.g. to conceptualise, to implement, to analyse). This highlights the need for more intensive supervision and mentoring, particularly for groups which show problems in aptitude and abilities during the workshop. Notably, access to peer-reviewed international publication had not been identified as a major obstacle. This may reflect the increasing availability of open access scientific publications in the past decade as well as the support of TORG mentors, which include, if necessary, assisting in obtaining essential literatures.

The performance of Indonesia's NTP has been exemplary in recent years as highlighted in the Joint External Monitoring Mission 2011 Report (unpublished) and the Joint External Monitoring Mission 2013 Report (unpublished), but it is difficult to actually attribute the contribution of OR to programmatic performance as there are so many other contributing factors (e.g. human resources, logistics, financing, and political commitment) and there are also challenges of measurement (15). The Joint External Monitoring Mission Report 2013 also highlighted that not all OR results have been utilised optimally for policy.

Assertions of limited impact to policy were apparently based on limited existing information because of lack of previous efforts to actually document policy uptake. Preliminary findings from an ongoing study led by Probandari et al. (personal communication) suggest that the recommendations of at least half of the OR groups supported by TORG have been taken up in TB control policies to various extents. This has been attributed to the intensity of communications between the researchers, programme managers, and policy makers facilitated through the capacity building process. However, the high turnover of programme managers and policy makers in the provincial/district level has seemingly been a major bottleneck to long-term relationships and policy influence. Notwithstanding, TORG has recently introduced efforts in line with current concepts and good practices to improve utilisation of research results in policy making (20–22). TORG members have also contributed substantially to some key policy documents produced by NTP and policy recommendation exercises. Evidently, TORG has become part of the TB policy network in Indonesia with considerable political capital within NTP. Such close collaborations and relationships with policy makers have been reported to be among the most important factors in influencing the use of evidence (23). Strong links to policy makers apparently facilitates trust and influence (22). Continuous and close personal contacts between researchers and programme managers are key in building such links (24). The context of rapidly increasing funding and scaling up of disease control services apparently also presents fertile ground for researchers to engage with policy makers and practitioners (25).

The influence of OR on (evidence-based) policy building might vary across the country, because Indonesia has a highly decentralised health system with political responsibility down to district level (20). There is a need to systematically document how the results of OR actually affect policy making at district level in decentralised health systems. Notwithstanding, the promotion of closer relationships between academicians and TB programme officers at provincial and district levels built through the workshops suggests that some impact on policy making can also be expected at this level (15, 21, 23). More leverage would still be needed, which perhaps can be gained through innovative efforts such as implementation of workshops to translate evidence into policy.

There are other aspects of research impacts which have been proposed to be documented (26). However, it is challenging for any research initiative to come up with coherent and comprehensive narratives of research impacts (15). In many cases, this will need specialised impact assessment studies requiring resources and skills which are not readily available. Such impact assessment is much needed and should be included in future OR capacity building efforts. In the meanwhile, embedding OR in national programmes should be a key priority for those aiming to alleviate the burden of diseases of public health importance in LMICs.

Most importantly, TORG and similar initiatives to embed OR into disease control programme should move forward while taking stock of the key reflections from the years of experience, including: ensuring long-term perspective and commitment of key stakeholders; mobilising external technical and financial assistance to address critical gaps in local resources and capacities; instilling continuous learning for improvement and adaptation; identifying strategies for sustainability; demonstrating tangible results and practical impacts; and integrating OR indicators into the set of key performance indicators of the national disease control programme.
